# Managing vesicoureteral reflux in children: making sense of all the data

**DOI:** 10.12688/f1000research.16534.1

**Published:** 2019-01-08

**Authors:** Angelena Edwards, Craig A. Peters

**Affiliations:** 1 Children’s Health System Texas, University of Texas Southwestern, Dallas, TX, USA

**Keywords:** Pediatric Vesicoureteral Reflux, Urinary tract infection, renal scarring, antibiotic prophylaxis

## Abstract

Current management of vesicoureteral reflux (VUR) in children is the result of a steady albeit controversial evolution of data and thinking related to the clinical impact of VUR and urinary tract infection (UTI) in children, the value of clinical screening, and the relative impact of testing and interventions for VUR. While controversy continues, there is consensus on the importance of bladder dysfunction on VUR outcomes, the likelihood of VUR resolution, and the fact that not all children with VUR require active treatment. Early efforts to define risk stratification hold the most promise to provide more patient-specific treatment of UTI and VUR in children.

## Introduction

The evolution of vesicoureteral reflux (VUR) management has been guided by changing understanding of the key elements of this common condition. Our perspective has changed as we see fewer children with the severe complications of reflux, arguably owing to more aggressive management. Due to this shift in clinical severity, reflux is seen in a more benign light. There is ongoing controversy as to how to balance the various elements of clinical reflux and their potential health impact. As we gain a better picture of the scope of VUR, we will need to define what is unacceptable morbidity and the clinical cost to prevent that morbidity. How much over-treatment will we accept to limit what level of under-treatment? This review will discuss the most recent studies and thinking in the area of VUR and how these trends are shaping our future care of these children and their families.

## Background

The true incidence and clinical significance of VUR in the general population are unknown, but the current reported values are impacted by sampling error and selection bias due to the fact that the majority of screenings and diagnoses of VUR are related to a patient’s history of urinary tract infections (UTIs). VUR can commonly be detected more so in females and uncircumcised males older than 1 year of age because of the higher rates of infections in these two populations. VUR is often diagnosed during the evaluation of antenatal hydronephrosis, which now includes a voiding cystourethrogram (VCUG) as part of the management algorithm for patients with ultrasound findings of high-grade—Society of Fetal Ultrasound (SFU) grade 3 to 4—hydronephrosis, bilateral hydronephrosis, ureteral dilation, or concern for anatomical abnormalities. This is a cultural shift in evaluation that may lead to a change in the reported prevalence of VUR.

A meta-analysis of over 250 articles revealed that the prevalence of reflux was 31.1% in children who were evaluated for a UTI and 17.2% in those with normal kidneys who had VCUG for other indications, such as the diagnosis of hydronephrosis ^1^. There is a gender dichotomy shown by a higher prevalence in males at younger ages that becomes more prominent in females as a population ages. This was recently shown by Capozza *et al*. with a male-to-female prevalence of VUR of 3:1 in infants until 6 months of age and a shift that occurs at 21 to 24 months that shows an equal prevalence of VUR for both genders ^2^. This is in contrast to the marked female (92%) to male (8%) ratio as seen in the Randomized Intervention for Children with Vesicoureteral Reflux (RIVUR) trial ^3^.

The definition of what constitutes clinically meaningful VUR has developed into the major point of interest when it comes to screening and treatment. It is now recognized that there is a potential harm of diagnosis and intervening/surveying patients who may not have had any true clinical sequelae of their VUR diagnosis other than exposure to antibiotics, invasive imaging, parent and patient anxiety, and potential surgical intervention. This is weighed against the potential risk of missing the opportunity to diagnose VUR and prevent infections, potentially saving patients from renal scarring. The concept of delaying VCUG in the workup following febrile UTI is now supported by the American Academy of Pediatrics (AAP) guidelines for patients from 2 to 24 months of age; this is a practice change from the historical workup for febrile UTI ^4^.

A contemporary stance may be to identify children with cortical scarring or congenital dysplasia and to follow these patients with the intent of preventing the development of VUR-related sequelae such as hypertension, pregnancy complications, UTI, or chronic kidney disease rather than to resolve their VUR. This is bolstered by screening with non-invasive imaging looking for cortical abnormalities or scarring prior to invasive testing with VCUG, in conjunction with confounding factors in the patient’s history, such as when recurrent UTIs and bladder and bowel dysfunction (BBD) are present. The presence of infections and BBD may be more clinically relevant to the patient’s overall outcome when compared with the grade of VUR itself. The future of VUR lies in the development of a risk assessment of each patient and may be rooted in categorizing BBD into a meaningful system.

## Diagnostic algorithms

### Defining urinary tract infection

In the majority of cases in children, VUR is diagnosed after a febrile UTI episode or abnormality seen on ultrasound imaging, potentially more common in the prenatal hydronephrosis population, leading to a VCUG being conducted. Attention to detail on how a urine specimen was obtained and whether pyuria is present is key in the diagnosis of a UTI. Accurate diagnosis of UTI is a critical turning point in the overall algorithm for how the patient will be evaluated and guides major clinical decisions. These decisions often hinge on the presence of recurrent infection and may lead to repeat imaging or medical/surgical intervention.

In 2011, the AAP revised the guidelines for the diagnosis and management of initial UTI in febrile infants from 2 to 24 months. The guidelines stress the importance of appropriate urine specimen collection, and a diagnosis of UTI is made only when at least 50,000 colonies per milliliter of a single uropathogenic species is present along with pyuria. The specimens should be collected via catheterization or suprapubic aspirate and a urine culture sent. If the patient has a febrile UTI, he or she will receive a renal and bladder ultrasound; if there are no anatomical abnormalities, no further imaging is conducted at that time ^4^. Midstream urine collection from toilet-trained children is acceptable but should be carried out in males with foreskin retracted. These guidelines have led to a change in the threshold to try to make a diagnosis of VUR and VCUG is not automatically part of the initial febrile UTI workup unless anatomical abnormalities are seen on the screening ultrasound. This has led to a decrease in the number of VCUGs performed and a steady decline in procedures performed for VUR ^5^. To date, however, it does not appear to have enriched the yield of significant reflux in the populations being tested ^6^.

### Imaging evaluation

**Figure 1.**
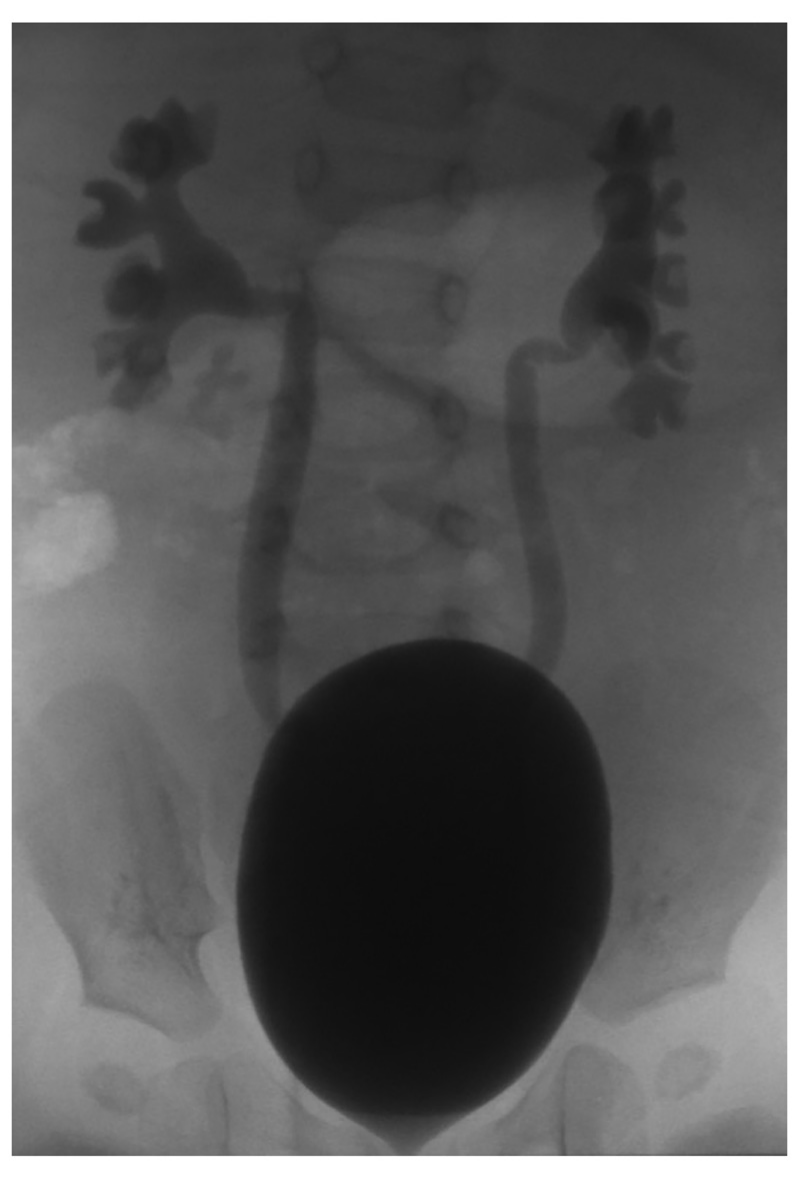
Voiding cystourethrogram showing bilateral grade 3 vesicoureteral reflux with a large, smooth-walled bladder. The calyceal configuration is normal without evidence of clubbing, which might have suggested renal parenchymal scarring. This is an original, unpublished image obtained by the author for this publication.

**Figure 2.**
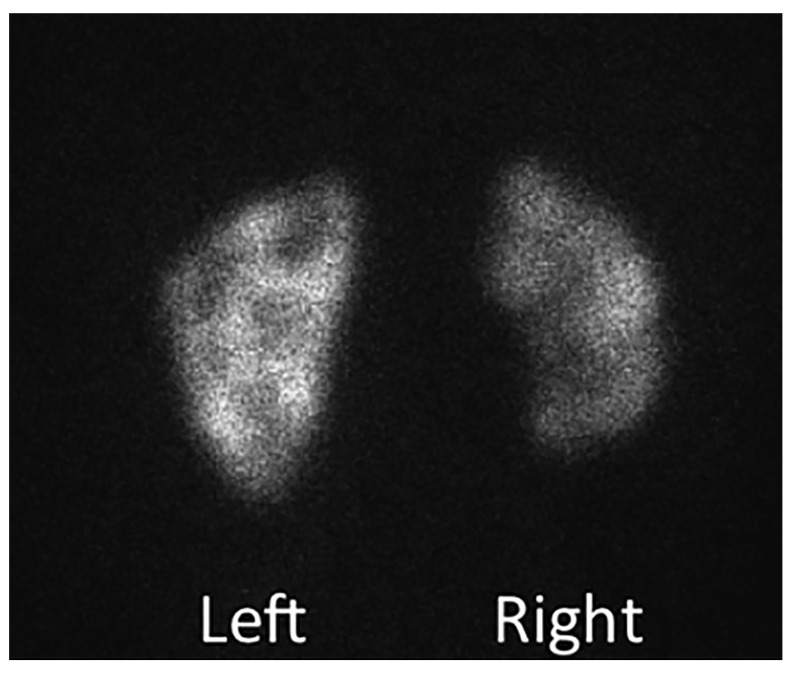
Dimercaptosuccinic acid scan showing significant right renal scarring and more focal cortical abnormalities on the left. This is an original, unpublished image obtained by the author for this publication.

The decision to perform voiding cystography, which is viewed as an invasive exam because of the need for catheterization, should be appropriately weighed with patient history, including BBD, recurrent infections, changes seen on renal ultrasound, and patient and parental desires. VCUG remains the gold standard, however, in terms of defining the presence, grade, and possible impact of VUR ( Figure 1). Ongoing attempts to standardize this study for modern usage are underway ^7^. The principal techniques used in the evaluation of the renal impact of VUR include dimercaptosuccinic acid (DMSA) scanning ( Figure 2) and renal ultrasound because of their lack of need for sedation or catheterization. However, DMSA scans may continue to become more limited because of accessibility and radiation exposure to the patient. Veenboer *et al*. showed that, out of 242 adult renal units evaluated with spinal dysraphism, DMSA scintigraphy could demonstrate more renal scars than ultrasound at a rate of 45.9% compared with 10.3% of renal units, demonstrating the greater sensitivity of DMSA scanning for renal scarring ^8^. The access to the tracer technetium-99m DMSA and need for peripheral intravenous placement have limited the use of renal scans in certain parts of the world in recent years. Ultrasound remains the most feasible upper tract imaging modality because it is the least invasive and most readily available. The ability to detect large cortical defects and renal asymmetry or discrepancies in renal growth makes it a reasonable initial and follow-up imaging modality.

This approach of imaging the upper tract prior to VCUG was popularized as the “top-down approach” (TDA). TDA operates under the assumption that clinically significant VUR will have associated changes on acute DMSA renal scans and are therefore worthwhile to detect but assumes that in patients with normal DMSA scans any VUR is clinically insignificant. With this approach, a patient receives a DMSA scan shortly after a UTI episode and if abnormalities are present this is followed up with VCUG. Preda *et al*. prospectively evaluated the TDA in 290 patients younger than 1 year of age who presented with UTI (79% febrile infections) ^9^. This revealed that 51% of patients had an abnormal DMSA scan; of those, 85% (44 out of 52) were found to have VUR. Of the remaining patients, 8 were found to have VUR with a normal DMSA: low-grade VUR was diagnosed in 7 and grade 3 VUR in 1 male ^9^. This has been countered by a Cochrane Review conducted in 2016 by Shaikh *et al*., who concluded that DMSA lacks the accuracy to detect VUR of any grade and should be challenged as a screening test because of the high proportion of children labeled at-risk for high-grade VUR ^10^. Interestingly, this study found that children with a negative DMSA scan have a less than 1% probability of having high-grade VUR ^10^.

Numerous other novel markers—such as delta neutrophil index, neutrophil-to-lymphocyte ratio, D-dimer, erythrocyte sedimentation rate, procalcitonin, and C-reactive protein—have been introduced as potential triggers for imaging when evaluating patients with UTI. Shaikh *et al*. conducted a randomized controlled trial of 309 children with their first febrile UTI from age 1 to 24 months who underwent either a biomarker screening as the threshold or DMSA changes to trigger a VCUG ^11^. It was shown that top-down and biomarker-based screening for VUR had poor sensitivity, as they did not detect 33% and 29% of high-grade VUR, respectively ^11^.

### Evolution of vesicoureteral reflux management

The overarching goal of all VUR management is to prevent UTI and renal scarring by using the least invasive means to preserve maximum renal function and prevent hypertension. This becomes very multifactorial once you add parental and practitioner bias along with the length of time the patient has been diagnosed and treated. VUR management has evolved to include medical management or observation as the first choice for most reflux patients, and the approach must be individualized to focus on preventing UTI instead of only VUR resolution. This is demonstrated by the high likelihood of spontaneous resolution of reflux with correction of BBD, which decreases infections. It is broadly accepted, with some caveats, that sterile reflux does not pose any real harm to the kidneys. All treatment modalities should be considered when developing an individualized treatment plan based on patients’ risk stratification, compliance of the family, and access to care among other considerations. The overall clinical picture may lead providers to offer definitive correction in certain patients over medical management or active surveillance.

In consideration of the AAP guidelines, patients may not be diagnosed until after two pyelonephritic episodes or may be diagnosed at a more advanced age with renal scarring already present. In these patients, providers may be more inclined to encourage definitive treatment, rather than observation or continuous antibiotic prophylaxis (CAP) prior to another pyelonephritic episode. On the other end of the spectrum, good results have been shown by providers who are starting to wean off CAP therapy in toilet-trained children with resolution of BBD combined with close observation for the development of constipation or infections ^12^. The idea of active surveillance or cessation of CAP reinforces the need to prospectively risk-stratify the patients who are at risk for breakthrough UTI on CAP. Hidas *et al*. showed that patients who presented after a UTI episode, were female, or had high-grade (4 and 5) VUR are the most concerning populations ^13^. Identifying those patients at risk for recurrent UTI when taken off prophylaxis is an even more important group, as they pose a real and challenging risk.

## Spontaneous resolution

The discussion focuses on viewing VUR as a generally self-resolving condition and needing to define at what point it becomes a true pathologic condition. VUR has been shown to resolve spontaneously 68% of the time but with higher rates of resolution in patients with grade 1 to 3 VUR and at a more rapid pace ^14^. The rate of resolution of VUR not only is a factor of VUR grade at presentation but also may be related to BBD and its management ^15^. The use of active surveillance without prophylaxis could be an option for some patients, but it would be prudent to offer this option to families that are compliant and able to access care when symptoms of UTI arise. It was recently shown that a delay in the initiation of antibiotic therapy of more than 48 hours for febrile UTI leads to a 47% increased risk of developing renal scarring ^16^. Estrada *et al*. conducted a multivariate analysis of 2,462 children with primary VUR and generated a nomogram for rates of spontaneous resolution of VUR on the basis of numerous factors, including reflux grade ^17^. The resolution rates by grade were as follows: 72% for grade 1, 61% for grade 2, 49% for grade 3, and % for grades 4/5. A major flaw was that a significant portion of the higher-risk patients underwent surgical intervention ^17^.

More recently, Kirsch *et al*. created a validated tool called the VUR index (VURI), which predicts the resolution for children younger than 2 years of age ^18^. This tool has found that high-grade VUR, ureteral anomalies, female gender, and reflux occurring during filling are associated with lower resolution or require a greater time to resolve ^18^. VURI has been validated for the pediatric population, including children with VUR diagnosed after the age of 24 months, and seems to be a good instrument for facilitating individualized patient care and discussions with patients’ families ^19, 20^. Being able to establish appropriate risk stratification could be the key to minimizing patient discomfort and burden but also protecting them from the sequelae of renal scarring.

## Continuous antibiotic prophylaxis

VUR is not thought to cause UTI but potentially decreases the time for progression from cystitis to pyelonephritis. This could be related to numerous mechanisms but is potentially escalated by the endotoxin effects from bacteria on ureteral atony, leading to a decreased rate of clearance of bacteria from the upper tract ^21^. In patients with dilating VUR, stasis of urine in the collecting system, hydronephrosis, or ureteral dilation may be another significant risk for infections. The greatest risk for post-infectious scarring seems to occur within the first year of life, and the natural progression of VUR in infant males is different from the pattern seen in infant females ^2^. Several well-constructed trials have been carried out with the intent to define the role of CAP in the management of VUR, but no concise conclusion could be drawn from the data. The most encouraging outcomes from these trials are all of the questions that arise from their findings and the potential hope for future studies to shed light in the era of risk stratification of patients with VUR, allowing providers to determine what variables are important to include when assigning a risk classification to a patient.

## Major clinical trials in vesicoureteral reflux

### International reflux study in children

This study included children younger than 11 years old with high-grade VUR (grade 3 or 4) who presented with UTI and were randomly assigned to CAP or corrective open surgery with an American ^22^ and a European ^23^ arm. The European arm also recorded the presence of BBD in the patient population (18%), and the BBD, when untreated, led to more UTI and longer persistence of VUR along with grade variation during follow-up ^24^. Some of the surgically managed patients had complications with post-operative obstruction ^25, 26^ but overall had a decreased rate of pyelonephritis compared with CAP, and there was an equal incidence of UTI ^23^ in both groups and equal efficacy in reducing new renal scarring. At the 5-year follow-up, there was no significant difference between the surgically and medically managed groups in the development of new renal scarring or scar progression ^27^, but there were more new scars observed in patients who entered the study at a younger age or had parenchymal thinning present ^28^. This study therefore demonstrated the equivalence of medical and surgical therapy; however, the incidence of surgical obstruction reported in the European arm was higher than in the American arm and greater than in many other clinical reports.

### Swedish study

This trial is a multi-center prospective trial of 203 children (128 females and 75 males) from age 1 to 2 with dilating VUR grade 3 or 4 who received a VCUG and renal scan at entry in the study and then were randomly assigned to CAP (69), endoscopic treatment (66), or surveillance (68) ^29^. At 2 years of follow-up, all treatment arms showed reflux improvement with downgrading to grade 1 or 2 or resolution: prophylaxis group (39%), endoscopic group (71%), and surveillance (47%) improvement ^30^. There were 67 recurrent febrile UTIs in 42 females and 7 males. Females with recurrent febrile infections occurred in 19% of prophylaxis, 23% of endoscopic, and 57% of surveillance groups.

The authors concluded that females with recurrence of infection had a higher tendency to have persistent VUR after 2 years, but reflux grade at induction of study (whether grade 3 or 4) was not predictive of recurrence ^31^. A strong association with new renal damage and recurrent UTI was seen in the female population, all of whom were in the surveillance and endoscopically managed patient groups ^32^. The importance of this study is the inclusion of a surveillance cohort of patients along with an increased male patient population. BBD was demonstrated by urodynamic evaluation and was linked to recurrent UTI and reflux persistence as well as new scarring.

### PRIVENT trial

In Australia, a placebo-controlled trial with trimethoprim-sulfamethoxazole (TMP-SMX) was conducted with CAP for children younger than 18 years of age (median age was 14 months and 53% had grade 3 VUR or higher). Not all patients had VUR. A 6% decrease in UTI occurrence was seen in the CAP group over the placebo, and the Bactrim-receiving patients had a UTI rate of 13% (36 out of 288) and the placebo group a rate of 19% (55 out of 288) ^33^. The authors concluded that CAP has a limited role, but the main criticism during this study was that no upper tract imaging was conducted to be able to comment on the presence of renal scarring between the two groups.

### RIVUR trial

RIVUR was a multi-center, double-blinded, randomized, placebo-controlled trial that evaluated 607 children (558 girls and 49 boys) who ranged in age from 2 to 71 months and who had grade 1 (11%), grade 2 (42%), grade 3 (38%), and grade 4 (8%) (grade 5 excluded) VUR that was diagnosed after UTI episode ^34, 35^. Patients were randomly assigned to placebo versus TMP-SMX antibiotic prophylaxis; this is very similar to the PRIVENT trial except that patients received DMSA at baseline and after 1 and 2 years of follow-up. After 2 years of follow-up, the CAP group had a 50% lower risk of UTI recurrence. However, 91.9% of the study population was female and VUR grade 1 to 3 was present in 91.7% of patients ^3^; therefore, the clinical significance has been challenged because of the attributes of the patient population, as it represents a relatively low-risk population. The most significant benefit with CAP was seen in those subgroups who had BBD or presented with a febrile UTI. DMSA scans at follow-up did not show any difference in new renal scarring between the two patient populations, although reduction in scarring was a secondary endpoint and the study was not powered to fully assess this feature. Although the reflux grading system used in the RIVUR trial has been used for decades, there was still relatively low inter-observer agreement on reflux grade. In the context of the study, three experts adjudicated grade, yet this observation would suggest that the use of reflux grade as a risk-stratifying factor may have significant clinical limitations ^36^. Recent investigations have suggested that using distal ureteral diameter as a “grading” scale for reflux severity may be more reliable than the traditional system ^37, 38^.

## Future directions

### Risk stratification

With regard to the future of VUR management, the best compromise is to identify the patients with the greatest risk of VUR sequelae and potentially never discover incidental VUR in a low-risk patient. A compelling concept is reclassifying the data from prior randomized controlled trials using risk classification and seeing whether the clinically significant risk factors can be identified with an acceptable level of morbidity. In 2018, the data from the RIVUR trial were re-evaluated using risk stratification ^39^. The clinical characteristics used to develop the risk stratification criteria are based on the model by Hidas *et al*. on UTI recurrence ^13^. Low-risk patients were defined as circumcised males with no BBD present and with grade 1 to 3 VUR. High-risk patients were classified as uncircumcised males with a grade 4 VUR or as females with BBD.

There were 385 (63.9%) patients in the low-risk population and 217 (36.1%) patients in the high-risk population for the RIVUR trial. Wang *et al*. showed that there is no significant difference in UTI recurrence in the low-risk group between CAP and placebo ( *p* = 0.151) but a higher UTI recurrence in the high-risk population and 31.5% reoccurrence in placebo versus 11.4% in CAP patients ( *p* = 0.001) ^39^. Patients in the high-risk cohort receiving placebo had a 3.7-fold increased risk of UTI recurrence over the 2-year period when compared with their CAP high-risk cohort, but no difference was found in renal scarring in the high- or low-risk grouping.

That no difference was seen in new or worsening renal scarring between the populations of this trial could be rooted in the fact that the majority of patients were female (91.9%) and had VUR grade 1 to 3 (91.7%) ^3^; therefore, the main characteristic affecting their risk stratification is BBD. To broadly apply the concept of standardized risk stratification to a more diverse population by combining the findings of the RIVUR and Swedish trials may lead to a more concrete understanding of risk stratification and the new frontier of VUR management.

### Bladder and bowel dysfunction

During the RIVUR trial, the dysfunctional voiding scoring system ^40^ was used to characterize patients. In the reclassification of the RIVUR data, patients who were not toilet-trained and had fewer than one bowel movement per day were categorized as having BBD. With these criteria, it was found during the reclassification that being a non-toilet-trained child with BBD was an independent predictor of breakthrough UTI in the CAP group ^39^.

**Figure 3.**
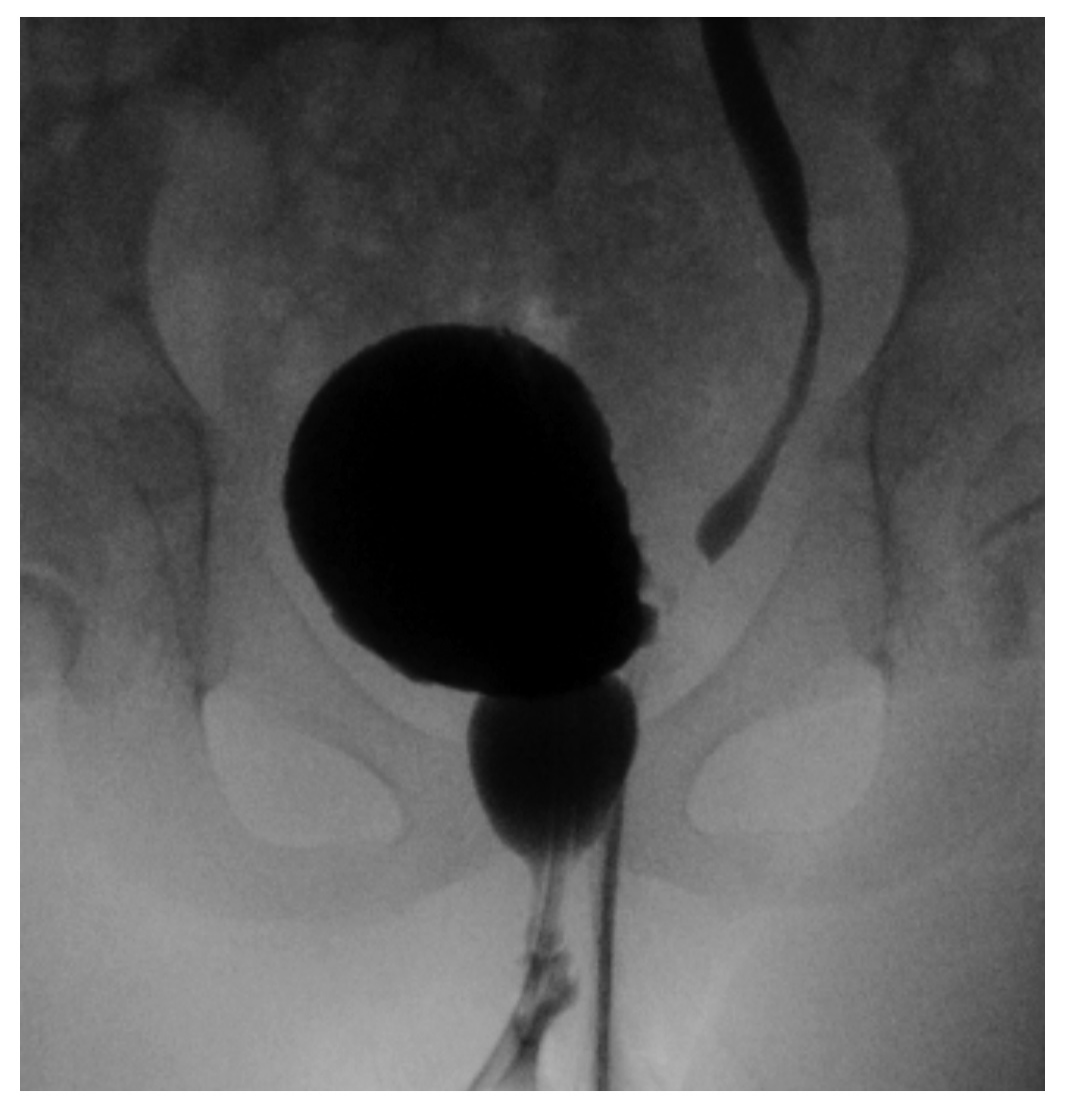
Voiding cystourethrogram showing left reflux as well as a dilated urethra in a girl with bladder and bowel dysfunction. This is the so-called “spinning top” urethra that results from incomplete relaxation of the external sphincter/pelvic floor. This is an original, unpublished image obtained by the author for this publication.

The importance of the management of BBD is emphasized in the 2010 meta-analysis conducted by the American Urological Association, which showed that BBD was associated with higher UTI incidence while on antibiotic prophylaxis, lower rates of resolution of VUR, and reduced success of endoscopic treatment of VUR ^41^ ( Figure 3). Children with VUR and coexisting BBD who underwent intervention with timed voiding, biofeedback, anticholinergic administration, or alpha blockers to address the lower urinary tract symptoms or voiding pattern were found to downgrade their reflux on average by two or more grades ^15^. In a study of 30 children with VUR, downgrading or resolution of VUR was seen in 70% of patients when treatment for BBD was incorporated into their treatment plan in the form of anticholinergic medication ^42^.

The incorporation of diagnosis and treatment of dysfunctional voiding and constipation into the initial evaluation of the patient could continue to improve the overall outcomes and shape areas of future research. Shaikh *et al*. were able to combine data from RIVUR and CUTIE (Careful Urinary Tract Infection Evaluation), which is a population of children younger than 6 years old who have had a UTI and were followed for 2 years ^43^. In this population, 54% of the 181 toilet-trained children had BBD (daytime incontinence, holding maneuvers, or constipation). Results showed a higher risk of developing UTI if VUR and BBD were both present when compared with children with VUR alone or BBD alone. Children with VUR and BBD were shown to have the greatest benefit from antibiotic prophylaxis ^43^.

## Surgery

The goal of reducing the recurrence of acute febrile UTI, renal scarring, and other complications of VUR can be fulfilled through surgery, which can be approached with cystoscopic subureteric injection, open ureteral reimplantation, and minimally invasive approaches such as robot-assisted laparoscopic ureteral reimplantation (RALUR). Patients who are more commonly considered for surgical management show recurrent UTI, deterioration of renal function, progression of renal scarring, complex anatomy, or parental/patient preference to avoid continuous antibiotic therapy and imaging.

### Endoscopic management

An option popularized over the last two decades is cystoscopic subureteric injection, which has been accomplished with several different materials over the years but currently is performed mostly with dextranomer/hyaluronic acid microspheres (Deflux). The goal of subureteric injection is to instill a bulking agent in the submucosal intramural tunnel in hopes of downgrading or resolving VUR. The endoscopic management has migrated from the subureteric Teflon injection (STING) technique (which originally used polytetrafluoroethylene injected below the ureteric orifice at the 6 o’clock position to generate a “crescent shaped” orifice) ^44^ to HIT (hydrodistention of the ureteric orifice and the injection of bulking agent is located in the mid to distal submucosal tunnel at the 6 o’clock position) ^45^ and now more commonly uses the double HIT technique (modified HIT technique with proximal and distal intraluminal submucosal injections) ^46^. A meta-analysis conducted by Yap *et al*. showed that the overall resolution of VUR was 82.5% with the HIT technique compared with 71.4% with the STING technique ( *p* <0.00001) ^47^. There are numerous publications on the success rate of VUR resolution by VUR grade, and the highest attainment was seen in lower grades of VUR ^48, 49^.

This technique, though an attractive option to avoid prolonged antibiotic exposure and invasive surgical treatments, may be limited by its durability over time. During the Swedish trial, the endoscopic cohort had recurrent dilating reflux in 20% of patients after 2 years as well as others with recurrence to grade 1 or 2 VUR ^30^. It should be noted that the STING procedure was used in the Swedish trial and initial success was very low. Currently reported success rates (cure of VUR or prevention of febrile UTI) using the double HIT method exceed 90%, and only 5% went on to open surgery after a single endoscopic treatment ^50^. Some may feel that the benefit of Deflux is to spare the patient from antibiotic prophylaxis while waiting for the natural resolution of VUR or coordinated maturation of voiding with toilet training to occur, even though its longevity over time is potentially inferior. The counterargument to this rationale is that the procedure requires general anesthesia and can take multiple injections, which come with their own concerns for patients.

National trends in VUR management were studied in 2014 by Herbst *et al*., who looked at 14,430 patients (17,826 procedures), of whom 49% underwent reimplantation and 51% received injection with dextranomer/hyaluronic acid from 2004 to 2011 in the PHIS (Pediatric Health Information System) database ^51^. The total number of VUR procedures has steadily declined through this time interval, but the average number of reimplantation surgeries remained steady, leading the author to conclude that this trend is declining for dextranomer/hyaluronic acid injections as a VUR treatment. However, in a similar study by Garcia-Roig *et al*. ^5^, the PHIS database was further evaluated to 2015, several years after publication of the 2011 AAP UTI guidelines. A total of 43,431 VCUG encounters and 28,484 anti-reflux procedures were evaluated (57% reimplantation, 41% dextranomer/hyaluronic acid injection, and 2% laparoscopic ureteral reimplantation). Following publication of the UTI guidelines, a significant overall drop in surgical intervention was noted for all procedure types. Although robotic surgical intervention comprised only 2% of all procedures, its utilization actually rose significantly after 2011 ^5^.

### Minimally invasive surgery

In the modern setting, patients and their families desire the least invasive option with the highest success rate and lowest complication rate; this lends itself to the current interest in RALUR. The introduction of RALUR in 2004 ^52^ has led multiple centers to adopt the technique, and most are able to achieve favorable outcomes approaching that of the gold-standard open-surgical approach ^53, 54^. There is a known steep learning curve to robotic surgery ^55^, and mastering the technique to match the reported outcomes is not universally experienced by all surgeons ^56, 57^.

The inherent benefits of robotic surgery are improved visualization and magnification, ergonomic comfort for the surgeon, cosmetic incisions ^58^, stabilization of movement with wristed instruments, shorter hospital stay ^59^, and decreased narcotic requirements ^60^. The downside is the increased cost ^61^ of robotic surgery and access to a robotic system along with a pediatric dedicated robot team ^62^.

With regard to current trends in VUR management, RALUR may not quickly replace the gold-standard open-surgical approach but is currently a viable option for patients presenting to surgeons with a robotic skillset and a dedicated robotics program. Some providers contend that RALUR has the most promise in caring for patients who are older or have complex anatomy ^63^. The future benefit of the robotic approach may lie in defining the patient characteristics that lead to a successful outcome ^64^ and may be able to be tied into the pre-operative risk stratification or anatomical considerations.

## Conclusions

Although it is unlikely that the controversies surrounding the evaluation and management of VUR will be resolved in the near future, the key trends are clear. With more robust patient risk stratification, those needing aggressive evaluation and subsequent management will be more clearly identified. The benefit will be fewer children receiving unproductive evaluations and imaging tests and more efficient identification of those in need of therapy. More refined imaging techniques are emerging to further support this evolution with reductions in morbidity. Continued development and improved patient selection for minimally invasive intervention will likely yield efficient and effective resolution of reflux and its risks.

## Abbreviations

AAP, American Academy of Pediatrics; BBD, bladder and bowel dysfunction; CAP, continuous antibiotic prophylaxis; DMSA, dimercaptosuccinic acid; HIT, hydrodistention-implantation technique; PHIS, Pediatric Health Information System; RALUR, robot-assisted laparoscopic ureteral reimplantation; RIVUR, Randomized Intervention for Children With Vesicoureteral Reflux; STING, subureteric Teflon injection; TDA, top-down approach; TMP-SMX, trimethoprim-sulfamethoxazole; UTI, urinary tract infection; VCUG, voiding cystourethrogram; VUR, vesicoureteral reflux; VURI, vesicoureteral reflux index
